# Neurogliaform cells mediate feedback inhibition in the medial entorhinal cortex

**DOI:** 10.3389/fnana.2022.779390

**Published:** 2022-08-08

**Authors:** Szilard Szocs, Nora Henn-Mike, Agnes Agocs-Laboda, Edina Szabo-Meleg, Csaba Varga

**Affiliations:** ^1^Szentágothai Research Centre, Department of Physiology, Medical School, University of Pécs, Pécs, Hungary; ^2^Department of Biophysics, Medical School, University of Pécs, Pécs, Hungary

**Keywords:** entorhinal cortex, feedback inhibition, GABA, neurogliaform cells, microcircuit, optogenetics

## Abstract

Layer I of the medial entorhinal cortex (MEC) contains converging axons from several brain areas and dendritic tufts originating from principal cells located in multiple layers. Moreover, specific GABAergic interneurons are also located in the area, but their inputs, outputs, and effect on local network events remain elusive. Neurogliaform cells are the most frequent and critically positioned inhibitory neurons in layer I. They are considered to conduct feed-forward inhibition *via* GABAA and GABAB receptors on pyramidal cells located in several cortical areas. Using optogenetic experiments, we showed that layer I neurogliaform cells receive excitatory inputs from layer II pyramidal cells, thereby playing a critical role in local feedback inhibition in the MEC. We also found that neurogliaform cells are evenly distributed in layer I and do not correlate with the previously described compartmentalization (“cell islands”) of layer II. We concluded that the activity of neurogliaform cells in layer I is largely set by layer II pyramidal cells through excitatory synapses, potentially inhibiting the apical dendrites of all types of principal cells in the MEC.

## Introduction

The medial entorhinal cortex (MEC) contains several functional neuronal types involved in spatial navigation, including grid cells, head direction, and speed cells (Hafting et al., 2005; Tukker et al., 2021). These cells anatomically and functionally belong to the glutamatergic/excitatory cell types that are localized in layers II–VI (Canto and Witter, 2012). The majority of the grid cells, however, are localized in layer II (Hafting et al., 2005), while the pyramidal cells in deeper layers (III–VI) code spatial information much less frequently. This layer specificity has attracted research on layer II, where MEC-specific microcircuit motifs have been described. For example, cell islands of calbindin+ pyramidal cells in layer II receive distinct perisomatic inhibitory inputs (Varga et al., 2010). Stellate cells in layer II have been shown to communicate with each other solely *via* fast spiking parvalbumin+ interneurons (Couey et al., 2013), meanwhile, layer II pyramidal cells have a higher connection probability with many surrounding neuronal types (Winterer et al., 2017; Zutshi et al., 2018).

The role of local GABAergic inhibitory neurons in generating the entorhinal cortex-specific cell activities is still not entirely known. Several studies focused on the function of parvalbumin+ fast-spiking interneurons, and only limited data have been published on the connectivity matrix of many other GABAergic cell types (Couey et al., 2013; Buetfering et al., 2014; Fuchs et al., 2016). Many interneurons are localized in layer I (Melzer et al., 2012), where apical dendrites of layer II–V pyramidal cells are located. The majority of these critically positioned interneurons are neurogliaform cells. Neurogliaform cells have been shown to elicit prolonged GABAA and GABAB receptor-mediated inhibition in the neocortex and hippocampus in virtually all cell types which are located within the range of the rich axonal clouds of the neurogliaform cells (Tamas et al., 2003). They are generally supposed to perform only feed-forward inhibition but no feedback inhibition. This phenomenon might be due to their somatodendritic location. In the somatosensory cortex, they are mostly in layer I where thalamic inputs excite them (Hay et al., 2021; Ibrahim et al., 2021). In the dentate gyrus, neurogliaform cells are positioned in the molecular layer where they receive excitatory entorhinal inputs (Armstrong et al., 2011). In the CA1 region, their dendrites extend only to the lacunosum moleculare and stratum radiatum where they receive entorhinal and CA3 inputs, respectively (Price et al., 2005). All these locations of neurogliaform cells, however, predicted that they do not receive local recurrent excitatory inputs (Wozny and Williams, 2011).

In this study, we aimed to shed light on the involvement of layer I GABAergic interneurons in the local microcircuits. Specifically, we investigated whether these cells receive excitatory inputs from the layer II pyramidal cells and whether neurogliaform cells show correlation to the island-like “patchy” structures which is a hallmark of entorhinal cortex superficial layers. Our results showed a strong, monosynaptic excitatory connection between layer II pyramidal cells and layer I interneurons. Therefore, we hypothesized that neurogliaform cells are involved in effective feedback inhibition of the layer II pyramidal cells. Moreover, we found that the neurogliaform cells are evenly distributed in layer I; therefore, they can elicit inhibition in all cell types sending dendrites to layer I.

## Materials and methods

### Experimental animals

The experiments were approved by the Ethics Committee on Animal Research of Pécs, Hungary (license #: BA02/2000-21/2021). The animals used in our experiments were male and female 6–14-week-old BL6 (C57BL/6J; JAX: 000664, The Jackson Laboratory, *N* = 22) and Calbindin-Cre (Calb1-IRES2-Cre-D: B6;129S-Calb1tm2.1(cre)Hze/J (JAX:028532), *N* = 10) mice and 6–8 week-old Wistar rats (RjHan:WI, Janvier-Labs, *N* = 8). The animals were housed in a 12 h light/12 h dark cycle. Animal handling was performed according to the regulations of the European Community Council Directive and approved by the Local Ethics Committee.

### Viral injection

For viral injection, the animals were deeply anesthetized (isoflurane, 4% initial dose for induction then 1% during the surgery). A small craniotomy was drilled in the skull above the MEC (coordinates were 3.75 mm lateral from the midline and 0.2 mm anterior to the transverse sinus). To selectively express ChR2 in Calb+ neurons, adeno-associated virus vector coding ChR2-mCherry fusion protein under the CBA promoter (AAV9.EF1.dflox.hChR2(H134R)-mCherry.WPRE.hGH (Addgene 20297), Penn Vector Core, University of Pennsylvania, United States) was injected 2.5–3.5 mm ventral from the craniotomy (40–70 nl of undiluted, ∼10^12^ GC/ml) at postnatal day P25–30 into the MEC. Calb-Cre mice were sacrificed 2 weeks postinjection for slice preparation.

### Slice preparation

Experiments were performed in acute horizontal brain slices taken from BL6 and Calbindin-Cre mice and Wistar Rats. Under deep isoflurane anesthesia, mice were decapitated, and 300 μm horizontal slices for MEC were cut in an ice-cold cutting solution containing: 85 mM NaCl, 75 mM sucrose, 2.5 mM KCl, 25 mM D-glucose, 1.25 NaH_2_PO_4_, 4 mM MgCl_2_, 0.5 mM CaCl_2_, and 24 mM NaHCO_3_ bubbled with 95% O_2_ and 5% CO_2_. After a 20 min incubation period at 34–37°C, the slices were transferred into artificial cerebrospinal fluid (ACSF) containing 2.5 mM KCl, 10 mM D-glucose, 126 mM NaCl, 1.25 mM NaH_2_PO_4_, 2 mM MgCl_2_, 2 mM CaCl_2_, and 26 mM NaHCO_3_ bubbled with 95% O_2_ and 5% CO_2_. Before the recording session, the slices were kept at room temperature. After recordings, the slices were immersion fixed overnight (4% paraformaldehyde, 0.1% picric acid in 0.1 M phosphate buffer) and then resectioned into 60 μm thin sections for biocytin visualization and immunohistochemistry.

### *In vitro* electrophysiological experiments

Patch pipettes were pulled from borosilicate glass capillaries with filament (1.5 mm outer diameter and 1.1 mm inner diameter; Sutter Instruments) with a resistance of 3–5 MΩ. Slices were visualized using an upright microscope (Nikon Eclipse FN-1 with 40×, 0.8 NA water immersion objective lens) equipped with differential interference contrast (DIC) optics and fluorescence excitation source (CoolLED pE-300^white^). Light intensities were measured using the Hand-held Optical Meter (Model 1918-R, Newport). DIC and fluorescence images were captured using an Andor Zyla 5.5 sCMOS camera. Whole-cell recordings were performed using the MultiClamp 700A amplifier (Molecular Devices), and signals were low-pass filtered at 4 kHz and digitized at 20 kHz using an Axon Digidata 1550B digitizer (with Clampex 11.1, Molecular Devices). The pipette recording solution contained “CsCl containing – high chloride – intracell”: 90 mM potassium gluconate, 3.5 mM KCl, 10 mM HEPES, 2 mM ATP-Mg, 0.4 mM GTP-Na, 10 mM phosphocreatine, 1.8 mM NaCl, 40 mM CsCl, 1.7 mM MgCl_2_, and 0.05 mM EGTA; “Low Cl containing intracell”: 130 mM potassium gluconate, 10 mM KCl, 10 mM HEPES, 0.2 mM EGTA, 2 mM ATP-Na, and 1.8 mM NaCl; “Very Low Cl containing intracell”: 135 mM potassium gluconate, 5 mM KCl, 10 mM HEPES, 0.2 mM EGTA, 2 mM ATP-Na, 1.8 mM NaCl, and 0.2% Biocytin, pH 7.3 adjusted with KOH, osmolarity: 290–300 mOsm. In some experiments, 10 μM NBQX (NBQX disodium salt hydrate; CAS 118876-58-7; N183, Sigma-Aldrich), 10 μM CNQX (Cat. No. 0190, Tocris), 1 μM TTX (Tetrodotoxin, Cat. No. 1078, Tocris), and 4-aminopyridine (CAS 504-24-5; 275875, Sigma-Aldrich) were used. Drugs were dissolved and stored according to the manufacturer’s instructions and were bath-applied. The light (full field, 490 nm) was flashed on the slices through the immersion objective lens. To normalize the ChR2 expression level variability between animals and slices, the light power was manually adjusted. *In vitro* data analysis was performed with the help of Clampfit 11.1 (Molecular Devices), Origin 9.5 (OriginLab Corporation), and the use of a custom script written in MatLab for determining the firing frequency of the cells.

### Immunohistochemistry, confocal microscopy, and Neurolucida-tracing

For immunostaining, animals were deeply anesthetized and transcardially perfused first with saline and then with 4% paraformaldehyde dissolved in 0.1 M phosphate buffer (PB, pH = 7.4). After overnight immersion postfixation, perfused brains and immersion fixed acute slices were sectioned into 40 μm tangential and 60 μm horizontal sections, respectively (Leica, VS1200s).

For the visualization of the biocytin-filled neurons, slices were incubated within Alexa 488-conjugated streptavidin (1:500) dissolved in 0.1 M PB solution for at least 5 h. The selected sections were incubated overnight at room temperature in the following antibody solutions (diluted in 0.1 M PB): Wfs1 (rabbit, 1:3,000; Abcam, ab176909), Calbindin (guinea-pig, 1:3,000; Synaptic Systems, 214005), GABAARα1 (rabbit, 1:1,000, Alomone, AGA-001), Reelin (mouse, 1:500, Millipore, MAB5367), Parvalbumin (rabbit, 1:30,000, Swant, PV28), CB1 (rabbit, 1:1,000, ImmunoGenes, IMG.pAb001), and α-actinin (mouse, 1:1,000, Sigma-Aldrich, A7811). Fluorescent labeling of the primary antibodies was performed by incubating the slices in solutions of different fluorescent dye (Alexa 405/488/594/647) conjugated donkey secondary antibodies (Jackson ImmunoResearch) raised against the host species of the primary antibodies. Confocal images were taken using a Nikon Eclipse Ti2-E fluorescence confocal microscope with 10×, 20×, and 60× objectives and a Zeiss LSM710 confocal microscope with a 20× objective. Z-stacking and brightness/contrast adjustment of the digital images were performed using the ImageJ software. The neurobiotin-filled cells were then prepared for light-microscopic reconstructions. In brief, sections were developed using the avidin-biotinylated horseradish peroxidase method using 3-3′-diaminobenzidine (DAB, Sigma-Aldrich) as described previously (Kecskés et al., 2020). The three-dimensional reconstructions of axonal and dendritic arborization were performed using the Neurolucida software (MicroBrightField Bioscience) with a 60× 1.4 NA objective. GABAARα1 and Reelin immunostainings and countings were carried out on 40 μm thick mouse brain slices, and fluorescent confocal imaging was performed later. GABAARα1 positive and Reelin positive cells were counted in layer I of the MEC with the help of Neurolucida software. Cells were counted in 8 slices from two animals (4-4 each). For quantification of cell densities within and outside of the patch regions, 30-μm thick rat brain slices were used. Slices were immunostained with α-actinin and Wfs1 antibodies, and fluorescence images were taken with a 10× 0.45 NA objective. Cell density was counted using the FIJI software. Cell densities were defined as cells/mm^2^.

### Statistics and reproducibility

Recorded cells were divided into two main groups based on electrophysiological and morphological properties: neurogliaform cells (NGF; *n* = 34, *N* = 10, 33 slices) and non-neurogliaform cells (non-NGF; *n* = 14, *N* = 10, 13 slices). The non-NGF group contains 6 stellate cells, 3 layer III pyramidal cells, and 5 non-neurogliaform interneurons. For paired recording, we used 23 BL6 mice and 5 Wistar rats, and the cells were identified based on their electrophysiological properties. A total of 39 cell pairs were recorded. Statistics were performed using GraphPad Prism. Normalities of samples were tested using the D’Agostino–Pearson test or the Kolmogorov–Smirnov test (for small numbers). Samples were compared with unpaired *T*-test and non-normally distributed ones using the Kruskal–Wallis test. Data were presented as mean ± S.E.M. The animal and the cell numbers are presented as *N* and *n*, respectively. For rise and decay time, 10–90% of the rise and decay slope was measured.

## Results

**FIGURE 1:**
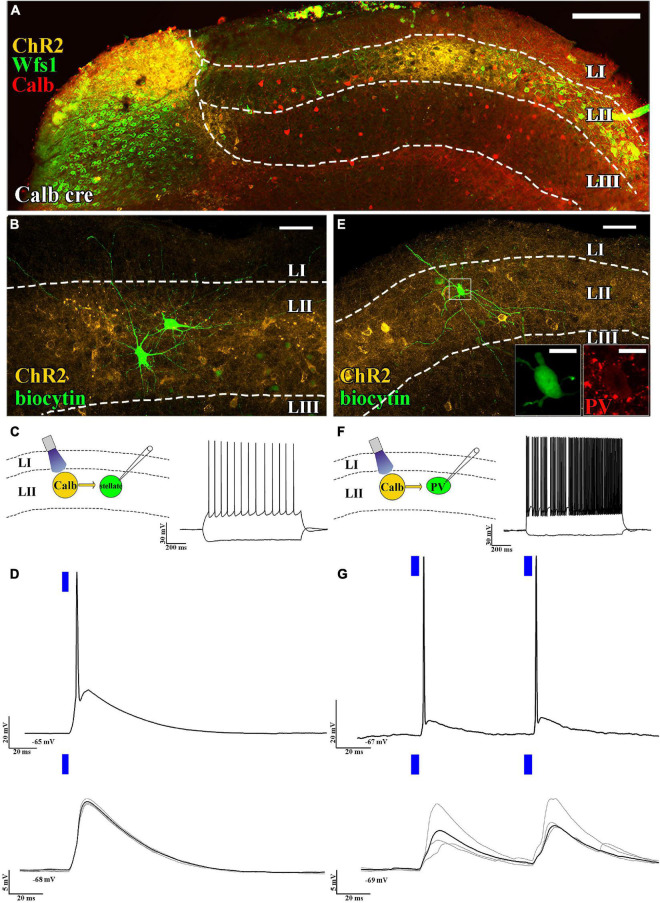
Axons of layer II pyramidal cells mainly target layer II. **(A)** Low-magnification confocal images of the horizontally sectioned MEC of a Calb-Cre mouse showing Cre-dependent local ChR2 expression (yellow). This ChR2 expression is colocalized with the specific LII pyramidal cell markers WFS1 (green) and calbindin (red) (scale bars: 200 μm). **(B)** High-magnification confocal images of a biocytin-filled layer II stellate cell and the surrounding ChR2-mCherry-positive axons (yellow) in the MEC of a Calb-Cre mouse (scale bars: 50 μm). **(C)** Schematic representation of optogenetic recording setup (left) and response to hyperpolarizing and depolarizing current steps of the recorded stellate cells (RMP: –65 mV and –100 and +200 pA, above, scale bar: 30 mV and 200 ms). **(D)** Response of the stellate cell to a suprathreshold (5 mW, above) and a subthreshold (0.25 mW, below) single 5 ms (blue bar) light pulse (RMP: –65 mV and –68 mV, respectively, scale bar: 20 mV and 5 mV, respectively, 20 ms). The black trace represents the average, while the gray traces represent 4 consecutive sweeps. **(E)** High-magnification confocal image of a parvalbumin-positive (immunoreactivity shown in the inset, scale bar: 10 μm) cell and the surrounding ChR2-mCherry-positive axons (yellow) in the MEC of a Calb-Cre mouse (scale bars: 50 μm). **(F)** Optogenetic recording schematics (left) and response to hyperpolarizing and depolarizing current steps of the recorded parvalbumin-positive cell (RMP: –67 mV and –100 and +200 pA, scale bar: 30 mV and 200 ms). **(G)** Response of the parvalbumin-positive fast spiker interneuron to suprathreshold (5 mW, above) and a subthreshold (0.25 mW, below) paired 5 ms light pulses (RMP: –67 mV and –69 mV, respectively, scale bar: 20 mV, 5 mV, and 20 ms). The black trace represents the average, while the gray traces represent 4 consecutive sweeps. The delay between the two pulses was 60 ms (scale bar: 20 mV, 5 mV, and 20 ms).

The layer II principal cells have been shown to excite several functional cell types (grid cells, head direction cells, etc.) located mainly in layers II and III (Zutshi et al., 2018). Their axonal targets have been reported to be located in layer II and to some extent in deeper layers (Sürmeli et al., 2015). High connection probability from layer II pyramidal cells to stellate cells has been reported (Winterer et al., 2017). To test this, we selectively expressed ChR2 in pyramidal cells using Calb-Cre mice (Figure 1A) and examined the effect of short (5 ms) light pulses on layer II stellate cells. It is noted that stellate cells that do not express calbindin or WFS1 (Varga et al., 2010; Kitamura et al., 2014) have pronounced “sag” response, roughly equally sized primary dendrites, and prominent main axon descending to deeper layers (Figures 1B,C), which are hallmarks of stellate cells (Alonso and Klink, 1993). We found that large amplitude excitatory postsynaptic potentials (EPSPs) (delay time: 2.0 ± 0.2 ms, *n* = 6, average amplitude of EPSPs: 10.6 ± 2.9 mV, at 5 mW light intensity) could be elicited in the stellate cells, which frequently resulted in action potentials (APs; Figure 1D). We also tested whether layer II fast spiking parvalbumin-expressing interneurons are also innervated by axons of layer II pyramidal cells (Figures 1E,F). Previously fast-spiking interneurons have been shown to be innervated by stellate cells (Couey et al., 2013), but their innervation by layer II pyramidal cells has not been directly shown. We found strong excitation (delay: 2.2 ± 0.7 ms, average amplitude of EPSPs: 6.8 ± 2.1 mV at 0.5 mW light intensity) on layer II interneurons (*n* = 5, 2/2 tested for parvalbumin), which reliably elicited APs when paired-pulse light-excitation was applied (Figure 1G). We also recorded from layer III pyramidal cells and found comparable results with layer II stellate cell recordings (delay: 2.3 ± 0.1 ms, average amplitude of EPSPs: 10.3 ± 5.1 mV, *n* = 3, light intensity: 2.5 mW, not shown). It is noted that recordings in voltage clamp mode gave similar results (refer to Supplementary Figure 1).

**FIGURE 2:**
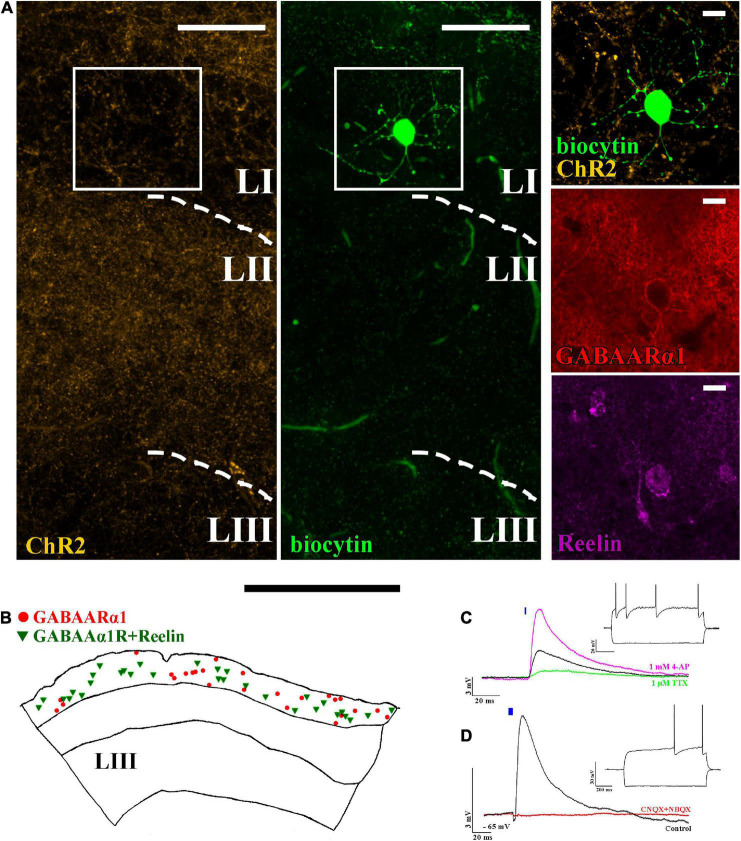
Layer I neurogliaform cells receive excitatory monosynaptic inputs from layer II pyramidal cells. **(A)** Confocal image of a biocytin-filled layer I neurogliaform cell surrounded by ChR2-mCherry-positive axons (yellow) (left). The recorded cell shows GABAARα1 (middle) and Reelin (right) immunopositivity (scale bars: 15 μm). **(B)** Representative reconstruction of somatic locations in a 40 μm thick section of the mouse medial entorhinal cortex showing layer I GABAARα1 (red circle) positive cells together with GABAARα1 and Reelin positive cells (green triangle) (scale bar: 500 μm). **(C)** The postsynaptic effects seen on neurogliaform cells (black, firing pattern in inset) were reduced by 1 μM TTX (green). Of note, 1 mM 4-AP not only recovered but also increased the amplitude of the postsynaptic effect, indicating monosynaptic input [RMP: –65 mV, excitatory postsynaptic potential (EPSP) amplitude: before TTX application 3.1 ± 0.6 mV, after TTX application 0.3 ± 0.2 mV and after 4-AP application 9.8 ± 2.9 mV, *n* = 4]. **(D)** The voltage response of the neurogliaform cell to a 5 ms photo-stimulation (black, single sweep EPSP, *n* = 34) and the disappearance of the effect after 10 μM CNQX + 10 μM NBQX wash in (red) (RMP: –65 mV, scale bars: 3 mV and 20 ms). Inset: response of the recorded cell to 1 s current injection (RMP: –65 mV and –100 and +150 pA). Note that the blue bars on panel **(C)** and **(D)** represent the 5 ms long light-pulses.

ChR2 expressing projections are not restricted solely to layer II but can also be found in layer I (Figure 2A). Therefore, we then investigated the effect of light pulses on layer I located interneurons. The majority of interneurons are neurogliaform cells (Figure 2A), which express GABAARα1 (Armstrong et al., 2012) and Reelin (Figure 2). In our colocalization experiments, we found that 53 ± 12% (totally 735 Reelin and/or GABAARα1 + cells, *N* = 2) of GABAARα1 expressing cells in layer I also express Reelin (Figure 2B). Their dendrites and axons are mostly restricted to layer I (Figure 2A; Craig and McBain, 2015). Light excitation of ChR2+ axons elicited EPSPs in neurogliaform cells (Figure 2C, delay: 1.9 ± 0.1 ms, amplitude: 6.0 ± 0.7 mV, *n* = 34, light intensity: 5 mW). TTX and 4-AP wash-in experiments verified that the recorded EPSPs are monosynaptic (Figure 2C).

Calbindin is expressed not only in layer II pyramidal cells but also in some GABAergic interneurons (Canto et al., 2008), potentially resulting in a mixed GABAergic and glutamatergic effect of light-activation of calbindin+ ChR2-expressing cells in Calb-Cre animals. We, therefore, tested whether neurogliaform cells receive inputs from calbindin+ interneurons as well. For this, we used a high-chloride intracell solution, kept the membrane potentials at −60 mV (refer to the section “Materials and methods”), and washed the sections in NBQX and CNQX (both in 10 μM). The AMPA receptor blockers have completely removed the postsynaptic effect on the neurogliaform cells (before drug wash, the average amplitude of the EPSPs was 6.8 ± 1.5 mV, *n* = 3, light intensity: 5 mW). Since there was no further postsynaptic effect after blocking AMPA receptors, we assume that there is no GABAA receptor-mediated input to the neurogliaform cells from calbindin+ interneurons within the entorhinal cortex (Figure 2D).

**FIGURE 3:**
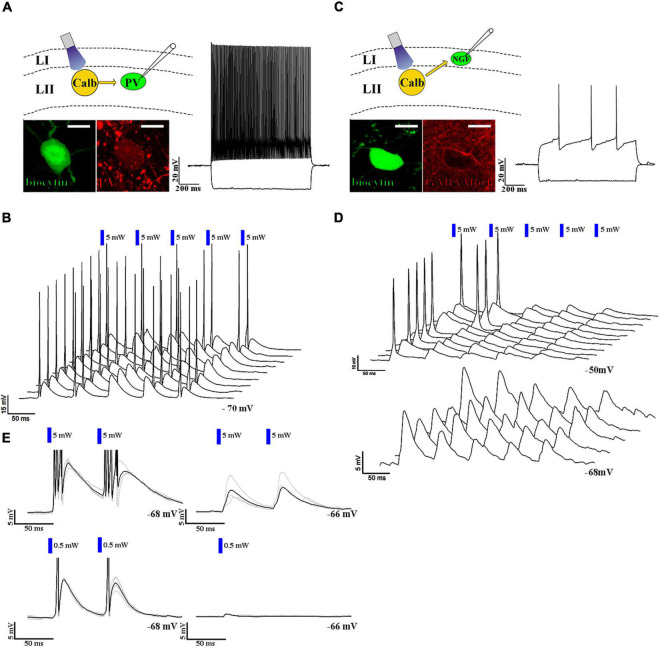
Neurogliaform and non-neurogliaform cells react differently to layer II excitatory inputs. **(A)** Up left: schematic representation of the recording setup. Bottom left: confocal image of biocytin-filled parvalbumin-positive fast-spiking interneuron soma (scale bar: 10 μm). Right: response to a 1 s current injection (RMP: –70 mV and –100 and +200 pA, scale bar: 20 mV and 200 ms). **(B)** 3D waterfall plot of the responses to five 5 ms long and 5 mW (blue bars) light pulses at 17 Hz. Sweeps were run 3 s apart. The last sweep in front (RMP: –70 mV, scale bars: 15 mV and 50 ms). **(C)** Up left: schematic representation of the recording setup. Bottom left: biocytin-filled GABAARα1 immunopositive neurogliaform cell body (scale bar: 10 μm). Right: response to a 1 s current injection (RMP: –68 mV and –100 and +200 pA). **(D)** 3D waterfall plot of the responses to five 5 ms long, 5 mW (blue bars) light pulses at 17 Hz at resting membrane potential (–68 mV, bottom), and at a depolarized state (–50 mV, top) (scale bars: top: 10 mV and 50 ms, bottom 5 mV and 50 ms). **(E)** Response of the parvalbumin-positive fast-spiking interneuron (left) and the neurogliaform cell (right) to paired 5 mW (above) and 0.5 mW (below) intense 5 ms light-pulses (RMP: –68 and –66 mV, respectively, scale bars: 5 mV and 50 ms). The black trace represents the average, while the gray traces represent 4 consecutive sweeps. The delay between the two pulses was 60 ms. The response of the non-neurogliaform interneuron to paired 5 ms long light pulses with 0.5 mW light intensity was 11.9 mV at RMP: –68 mV, while the response of the neurogliaform cell to a single 5 ms long light pulse with the same intensity was 0.7 mV at RMP: –66 mV. It is noted that the response of neurogliaform cell to a 0.5 mW light pulse was tested only with a single 5 ms long light pulse (right, below).

Neurogliaform cells are considered late-spiking cells (Kawaguchi, 1995), which means that when somatic membrane depolarization is applied to them, they elicit their first action potentials only after several hundred ms during the depolarization step (Figure 2D inset). The neurogliaform cells in the MEC show similar late-spiking intrinsic membrane properties (rheobase: −36.6 ± 0.9 mV, first AP after 278.5 ± 48.0 ms, resting membrane potential of NGF cells: −65.1 ± 0.5 mV, *n* = 34, Figure 2D inset; time constant: 7.5 ± 0.4 ms; Supplementary Figure 2). There is only limited data, however, about the EPSP-generated firing properties of this cell population (Chittajallu et al., 2020). Therefore, we then tested how neurogliaform cells and other non-late spiking interneurons react to trains of EPSPs generated by layer II pyramidal cells. Layer II interneurons (*n* = 5, Figures 3A,B) reliably elicited action potentials during the train of stimulation (5 EPSPs at 17 Hz, 10.7 ± 2.7 mV, light intensity 5 mW). In the layer I neurogliaform cells (Figure 3C), the same intensity of light elicited smaller amplitude EPSPs (average amplitude of EPSPs: 6.2 ± 1.1 mV, *n* = 17, light intensity 5 mW, Figure 3E) and could not elicit action potentials. However, when the membrane potentials were slightly depolarized from resting (from −64.5 ± 0.6 mV to −52.8 ± 0.9 mV), action potentials have been initiated. The occasional action potentials occurred during the first and/or second light pulses, and no action potentials could be elicited during the remaining 3 light pulses (Figure 3D).

**FIGURE 4:**
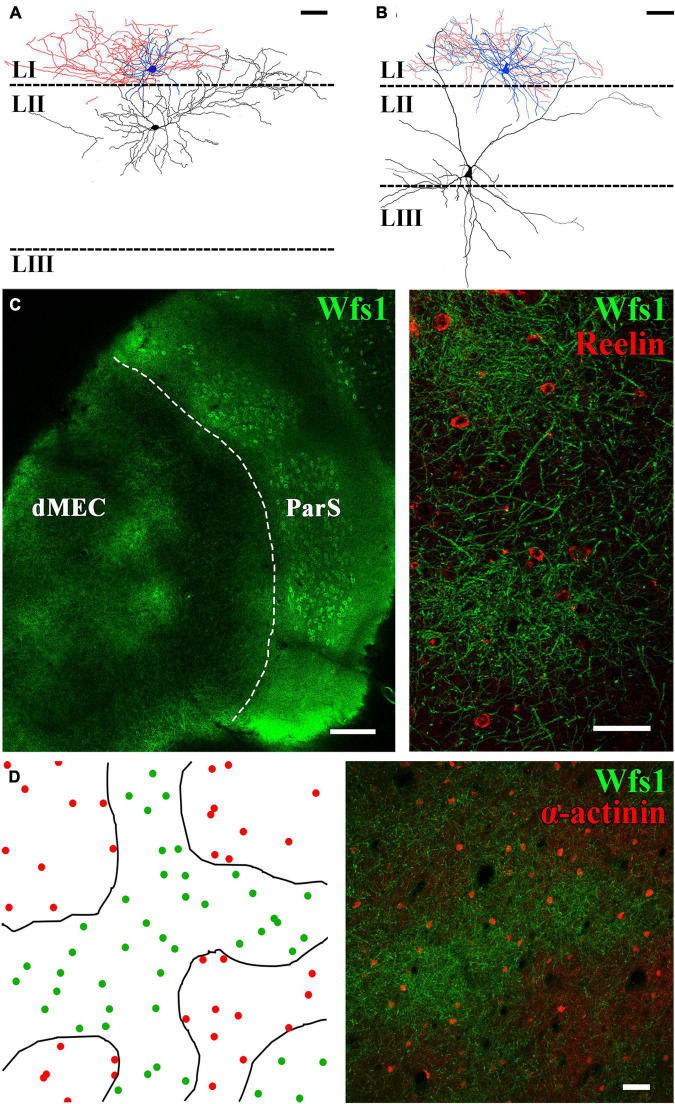
The distribution of neurogliaform cells does not correlate with dendritic clustering of layer II pyramidal cells. Neurolucida partial reconstruction of two paired recordings. **(A)** Neurogliaform cell (soma and dendrites blue, axons red) together with layer II pyramidal cell (soma and dendrites, black). **(B)** Neurogliaform cell together with layer II stellate cell. Neurogliaform cell dendrites and axons are mostly restricted to layer I (scale bar: 50 μm). **(C)** Tangential section of the mouse temporal cortex showing the dorsal MEC (dMEC) and the parasubiculum (ParS) (left, scale bar: 200 μm). In layer I, dendrites of layer II pyramidal cells form patches, and Reelin-positive interneurons are located within and in between patches as well (right) (z-stack of 7 μm section, scale bar: 50 μm). **(D)** Left: drawings of the patch borders (black lines) and the positions of α-actinin+ putative neurogliaform cells (green dots within patches and red dots outside patch structures) in the rat MEC layer I. Right: representative low-magnification confocal image of the dorsal MEC layer I, based on which the drawing was made (z-stack of 30 μm section, scale bar: 50 μm).

The connection probability between two cells has been shown to largely depend on the distance between the neurons (Holmgren et al., 2003). Following this assumption, we have attempted to record monosynaptic connectivity between layer II principal cells and closely located neurogliaform cells in control BL6 mice and Wistar rats (*n* = 15 stellate and *n* = 24 pyramidal cells recorded simultaneously with neurogliaform cells in layer I, right above the cell body of the layer II principal cell Figure 4). Since no connection has been found between the recorded cell pairs, we have analyzed their axo-somato-dendritic morphology both in rats and mice. The layer II pyramidal cells (sag potential at −100 pA: 1.2 ± 0.2, half-width of sag decay: 177.7 ± 7.3 ms, not shown) instead of having apical dendrites running straight up to layer I perpendicular to the layer, as layer II–III pyramidal cells in the neocortex (Feldman, 1984) and layer III pyramidal cells in entorhinal cortex have (Canto and Witter, 2012; Craig and McBain, 2015; Kecskés et al., 2020), MEC layer II calbindin+ pyramidal cells send their most prominent “apical” dendrites laterally (Figure 4A). The layer II stellate cells [sag potential at −100 pA: 2.7 ± 0.4 mV, half-width of sag decay: 58.4 ± 4.5 ms, the sag potential amplitude and half-width of the decay time are significantly different from layer II pyramidal cells (*p* = 0.0020^**^ and *p* < 0.0001^****^, both unpaired *T*-test with Welch’s correction), not shown] (Winterer et al., 2017), in contrast, have both laterally and medially running dendrites and mostly no apical dendrites running straight up to the layer I (Figure 4B). These features of the layer II principal cells set different connectivity rules compared with the known connection probability rule of thumbs in other cortical areas. Then, we checked whether neurogliaform cells are actually localized in areas of layer I where dendrites of the layer II pyramidal cells are less abundant. For this, we labeled tangentially sectioned layer I of the entorhinal cortex area for markers of layer II pyramidal and neurogliaform cells. Both medial and lateral entorhinal cortex layer II pyramidal cell bodies are organized into well-defined cell islands (Kitamura et al., 2014; Witter et al., 2017). Patchy structures are recognizable in layer I as well (Figures 4C,D); however, instead of the hexagonal arrangement of the patches, we have found amorph WFS1+ areas intermingled with putative layer III-V pyramidal cells apical dendrites (Figures 4C,D). We have estimated the density of neurogliaform cells in the patch and interpatch areas. Our semiquantitative measurement of alpha-actinin immunoreactive putative neurogliaform cells in rats (Ratzliff and Soltesz, 2001; Price et al., 2005) revealed that neurogliaform cells are relatively homogenously distributed within the patch and interpatch areas (within patches: 169 ± 24 cell/mm^2^ outside patches: 122 ± 5 cell/mm^2^, *p* = 0.1288 Welch corrected *t*-test, Figure 4D).

## Discussion

Using selective genetic markers of layer II pyramidal cells of the entorhinal cortex and optogenetics combined with slice electrophysiology, our results show that layer I neurogliaform GABAergic interneurons receive monosynaptic excitatory inputs from the layer II calbindin+ pyramidal cell. The layer II pyramidal cells in the MEC have been shown previously to target locally layers II and III (Winterer et al., 2017; Zutshi et al., 2018) and many other brain areas outside of the entorhinal cortex (Varga et al., 2010; Kitamura et al., 2014). Neurogliaform cells have been thought to be specialized to conduct feed-forward inhibition and receive no excitatory inputs from local glutamatergic cells. In the hippocampus, CA1 and dentate gyrus neurogliaform cells have been shown to be excited by entorhinal cortical input (Price et al., 2005; Armstrong et al., 2011) and in the neocortex by subcortical and long-projecting pyramidal cells from other cortical areas (Hay et al., 2021; Ibrahim et al., 2021). The local neocortical layer II–III pyramidal cells, dentate granule cells, and CA1 pyramidal cells have been, however, shown to avoid the innervation of neurogliaform cells (Wozny and Williams, 2011; Overstreet-Wadiche and McBain, 2015). In this study, therefore, we described an entorhinal cortex-specific circuit motif. In neocortical areas, layer I located apical dendrites and interneurons receive strong innervation from the thalamus; however, in the entorhinal cortex, there is no evidence for monosynaptic input from thalamic nuclei (Tukker et al., 2021). Thalamic innervation plays a critical role in the generation of upstates and downstates during sleep. The transition from depolarized and more active upstates to downstates is initialized by the thalamic innervation of neurogliaform cells in neocortical areas, which activate GABAB receptors on the apical dendrites of layer II–V pyramidal cells (Mann et al., 2009; Craig et al., 2013; Hay et al., 2021). The potential lack of direct thalamic innervation of neurogliaform cells in the entorhinal cortex predicts their alternative innervation. In this study, we have shown that the monosynaptic excitatory inputs from layer II pyramidal cells target layer I neurogliaform cells. This innervation by itself, however, was not sufficient to generate action potentials in the targeted neurogliaform cells during resting membrane potentials. When neurogliaform cells were slightly depolarized, which occurs during upstates (Craig and McBain, 2015), action potentials are generated mostly by the first EPSPs during a longer excitatory burst. The underlying mechanism of this firing pattern remained unresolved. Moreover, we have not investigated how neurogliaform cells react to the same excitatory protocol when hyperexcitable “barrage firing” state is induced (Chittajallu et al., 2020).

The termination of persistent firing has also been partially linked to GABAB receptor activation (Mann et al., 2009). In the entorhinal cortex deeper layer (III–V), cells show a tendency to sustain action potential trains (Egorov et al., 2002). Somatostatin+ interneurons have been shown to strongly inhibit *via* GABAA and somatostatin receptors on deeper layer pyramidal cells, without the activation of GABAB receptors (Kecskés et al., 2020). Therefore, we hypothesized that GABAB receptor activation may be driven by neurogliaform cells in the entorhinal cortex, similar to the hippocampus and neocortex (Tamas et al., 2003; Price et al., 2005; Overstreet-Wadiche and McBain, 2015).

Taken together, we have shown an intrinsic network motif of the entorhinal cortex, which highlights the importance of layer II pyramidal cells in local feedback inhibition. They not only activate surrounding layer II stellate and fast-spiker interneurons but influence the activity of neurogliaform cells, which are known to have a global inhibitory action on the surrounding neuronal network.

## Data availability statement

The raw data supporting the conclusions of this article will be made available by the authors, without undue reservation.

## Ethics statement

The animal study was reviewed and approved by the Ethics Committee on Animal Research of Pécs, Hungary.

## Author contributions

SS, NH-M, AA-L, and CV designed the experiments and wrote the manuscript. All authors collected and analyzed the data and commented on the manuscript.

## Supplementary material

SUPPLEMENTARY FIGURE 1 Comparison of light-evoked excitatory postsynaptic currents (EPSCs) and excitatory postsynaptic potentials (EPSPs) in neurogliaform, non-neurogliaform, and principal cells of the medial entorhinal cortex (MEC). **(A)** EPSCs seen on neurogliaform cells (top), principal cells (middle), and non-neurogliaform interneurons (bottom). Black: average of ten consecutive sweeps. Gray: individual sweeps. Inset: response to hyperpolarizing and depolarizing current steps of the recorded cells (−100 and +150 pA). **(B)** Statistical comparison of the recorded cell types. Top: EPSC rise times (neurogliaform cells: 2.7 ± 0.4 ms, *n* = 34; principal cells: 3.6 ± 0.9 ms, *n* = 9; non-neurogliaform interneurons: 2.6 ± 0.6 ms, *n* = 5). Bottom: EPSC decay times (neurogliaform cells: 22.5 ± 2.2 ms; principal cells: 22.7 ± 3.2 ms; non-neurogliaform interneurons: 17.4 ± 5.4 ms). **(C)** EPSPs on the same cells as on **(A)**. Ten superimposed consecutive traces are shown in gray and the average in black. **(D)** Statistical comparison of the recorded cell types. Top: rise time of the neurogliaform cells: 6.1 ± 0.8 ms; of the principal cells: 3.7 ± 0.8 ms; of the non-neurogliaform interneurons: 6.0 ± 2.5 ms, respectively. Bottom: decay time of the neurogliaform cells: 66.0 ± 6.6 ms; of the principal cells: 51.6 ± 9.0 ms; of the non-neurogliaform interneurons: 79.2 ± 22.5 ms, respectively.

SUPPLEMENTARY FIGURE 2 The light-intensity dependence of evoked PSPs and the basic electrophysiological properties of neurogliaform and non-neurogliaform interneurons in calbindin-ChR2 MEC. **(A)** The amplitudes of PSPs elicited by different light intensities in neurogliaform and non-neurogliaform interneurons. The non-neurogliaform interneurons (red triangles) showed strong excitation (resulting in APs) even at the lowest light intensities, therefore, no data available with stronger light. Data of individual neurons are connected with gray (neurogliaform cells) or red (non-neurogliaform interneurons) lines. **(B)** The rheobase of neurogliaform cells (black circles, −36.6 ± 0.9 mV, *n* = 34) and non-neurogliaform interneurons (black triangles, −47.3 ± 4.7 mV, *n* = 5, *p* = 0.0006^***^ with unpaired *T*-test). **(C)** The input resistance of neurogliaform cells (225.7 ± 14.2 MΩ, *n* = 34) and non-neurogliaform cells (226.2 ± 42.48 MΩ, *n* = 5, *p* = 0.9898 with unpaired *T*-test). **(D)** The resting membrane potential of the neurogliaform cells (black circles) and non-neurogliaform interneurons (black triangles) (−65.1 ± 0.5 mV, *n* = 34; −65 ± 1.5 mV, *n* = 5; respectively, *p* = 0.9326 with unpaired *T*-test). **(E)** Time constants of neurogliaform and non-neurogliaform cells (7.5 ± 0.4 ms, *n* = 34; 12.3 ± 2.5 ms, *n* = 5; respectively, *p* = 0.0366* with Kolmogorov–Smirnov test).
