# Supplementary figures and images for: Neurogliaform cells mediate feedback inhibition in the medial entorhinal cortex
Source: Front Neuroanat. 2022 Aug 8;16:779390. doi: 10.3389/fnana.2022.779390 (PMC9393258; doi:10.3389/fnana.2022.779390)

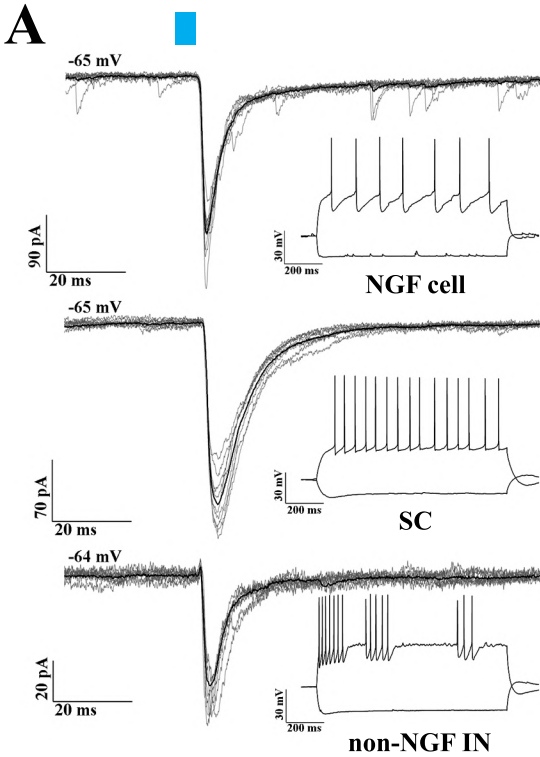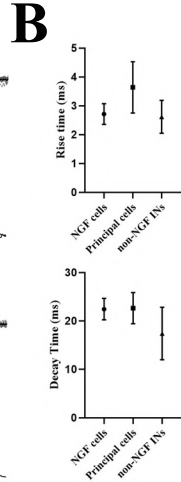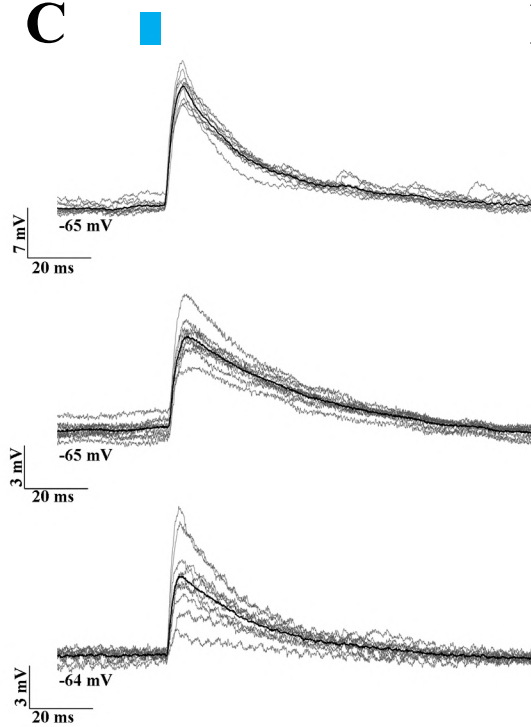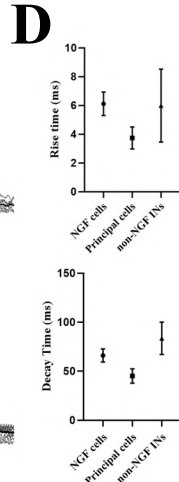

**A**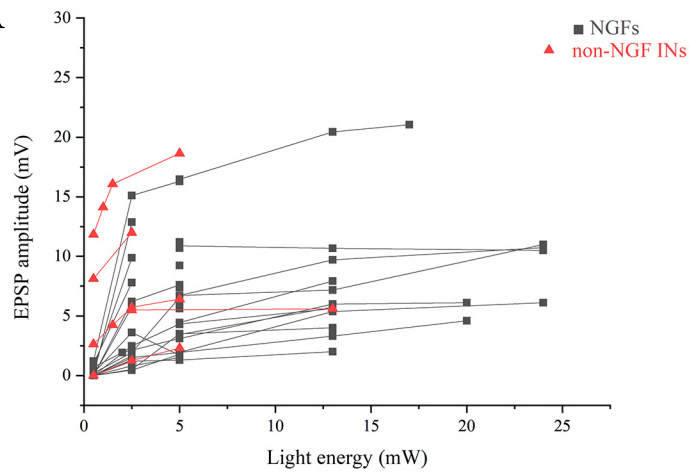**B**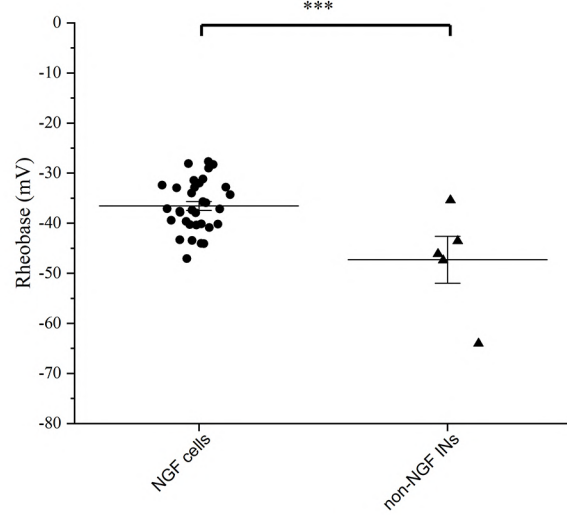**C**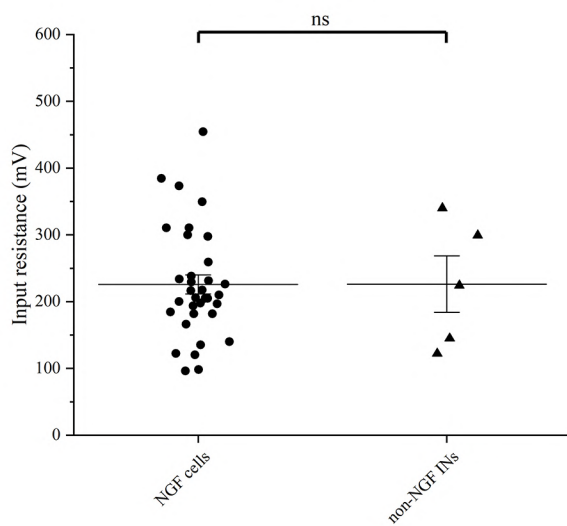**D**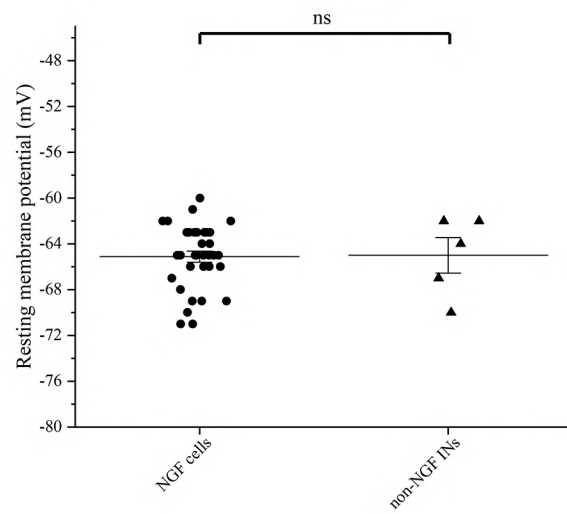**E**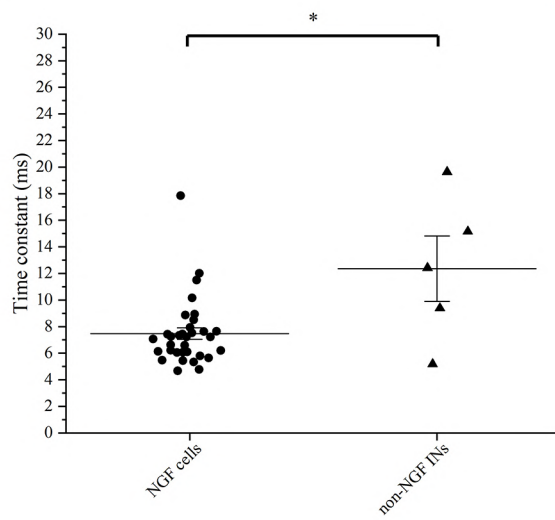

Supplement: SUPPLEMENTARY FIGURE 1 — Comparison of light-evoked excitatory postsynaptic currents (EPSCs) and excitatory postsynaptic potentials (EPSPs) in neurogliaform, non-neurogliaform, and principal cells of the medial entorhinal cortex (MEC). (A) EPSCs seen on neurogliaform cells (top), principal cells (middle), and non-neurogliaform interneurons (bottom). Black: average of ten consecutive sweeps. Gray: individual sweeps. Inset: response to hyperpolarizing and depolarizing current steps of the recorded cells (−100 and +150 pA). (B) Statistical comparison of the recorded cell types. Top: EPSC rise times (neurogliaform cells: 2.7 ± 0.4 ms, n = 34; principal cells: 3.6 ± 0.9 ms, n = 9; non-neurogliaform interneurons: 2.6 ± 0.6 ms, n = 5). Bottom: EPSC decay times (neurogliaform cells: 22.5 ± 2.2 ms; principal cells: 22.7 ± 3.2 ms; non-neurogliaform interneurons: 17.4 ± 5.4 ms). (C) EPSPs on the same cells as on (A). Ten superimposed consecutive traces are shown in gray and the average in black. (D) Statistical comparison of the recorded cell types. Top: rise time of the neurogliaform cells: 6.1 ± 0.8 ms; of the principal cells: 3.7 ± 0.8 ms; of the non-neurogliaform interneurons: 6.0 ± 2.5 ms, respectively. Bottom: decay time of the neurogliaform cells: 66.0 ± 6.6 ms; of the principal cells: 51.6 ± 9.0 ms; of the non-neurogliaform interneurons: 79.2 ± 22.5 ms, respectively. [file Data_Sheet_1.PDF]
